# Unravelling transcriptomic complexity in breast cancer through modulation of DARPP-32 expression and signalling pathways

**DOI:** 10.1038/s41598-023-48198-y

**Published:** 2023-11-30

**Authors:** Behnaz Saidy, Richa Vasan, Rosie Durant, Megan-Rose Greener, Adelynn Immanuel, Andrew R. Green, Emad Rakha, Ian Ellis, Graham Ball, Stewart G. Martin, Sarah J. Storr

**Affiliations:** 1 Nottingham Breast Cancer Research Centre, Biodiscovery Institute, School of Medicine, University of Nottingham, University Park, Nottingham, NG7 2RD UK; 2 Medical Technology Research Centre, Anglia Ruskin University, Bishop Hall Lane, Chelmsford, CM1 1SQ UK

**Keywords:** Breast cancer, Tumour biomarkers

## Abstract

DARPP-32 is a key regulator of protein-phosphatase-1 (PP-1) and protein kinase A (PKA), with its function dependent upon its phosphorylation state. We previously identified *DKK1* and *GRB7* as genes with linked expression using Artificial Neural Network (ANN) analysis; here, we determine protein expression in a large cohort of early-stage breast cancer patients. Low levels of DARPP-32 Threonine-34 phosphorylation and DKK1 expression were significantly associated with poor patient prognosis, while low levels of GRB7 expression were linked to better survival outcomes. To gain insight into mechanisms underlying these associations, we analysed the transcriptome of T47D breast cancer cells following DARPP-32 knockdown. We identified 202 differentially expressed transcripts and observed that some overlapped with genes implicated in the ANN analysis, including *PTK7*, *TRAF5*, and *KLK6*, amongst others. Furthermore, we found that treatment of DARPP-32 knockdown cells with 17β-estradiol or PKA inhibitor fragment (6–22) amide led to the differential expression of 193 and 181 transcripts respectively. These results underscore the importance of DARPP-32, a central molecular switch, and its downstream targets, DKK1 and GRB7 in breast cancer. The discovery of common genes identified by a combined patient/cell line transcriptomic approach provides insights into the molecular mechanisms underlying differential breast cancer prognosis and highlights potential targets for therapeutic intervention.

## Introduction

DARPP-32, first identified four decades ago, is expressed in dopamine innervated brain regions and is highly concentrated in the basal ganglia; it is encoded by *PPP1R1B*^1–3^. Its initial discovery was as a potent inhibitor of protein-phosphatase-1 (PP-1)^4^, and its inhibitory action was quickly found to be dependent on its phosphorylation status, mediated through cAMP-dependent protein kinase (PKA)^5^. Over time, DARPP-32 has been shown to play a pivotal role in regulating both biochemical and electrophysical responses to a range of physiological and pharmacological stimuli. Notably, DARPP-32 phosphorylation is modulated by dopamine, and numerous other neurotransmitters, and can also be influenced by drugs of abuse^6,7^. DARPP-32 acts as a bifunctional signaling protein that can either inhibit kinases or phosphatases dependent upon the phosphorylation of its principal phosphorylation sites, Threonine (Thr)-34 and Thr-75. Phosphorylation of DARPP-32 at Thr-34 by PKA results in potent inhibition of PP-1; while phosphorylation at Thr-75 by cyclin-dependent kinase 5 (Cdk5) inhibits PKA^8^.

DARPP-32 signaling, a vital molecular switch in the brain, has been implicated in cancer. Notably, truncated-DARPP (t-DARPP), a cancer-specific variant of DARPP-32 lacking the Thr-34 phosphorylation site, was originally described in gastric cancer^9^. Increased expression of both DARPP-32 and t-DARPP has been observed in breast, prostate, colon, and stomach cancer tissue when compared with normal tissue^10,11^. Furthermore, studies have indicated that DARPP-32 expression occurs after a phase of dysplasia in oesophageal squamous cell carcinomas, with tumours displaying DARPP-32 expression progressing at a slower pace than those without^12^. Expression levels of DARPP-32 and t-DARPP have been linked with tumour growth, patient survival, and response to treatment in lung cancer^13–16^ and gastric cancer^17–20^. The link between DARPP-32 and t-DARPP with tumourigenesis and response to treatment, also extends to breast tumours.

High levels of a combination of both DARPP-32 and t-DARPP expression have been linked with poor survival of breast cancer patients (n = 230)^21^ and t-DARPP expression has been shown to increase during mammary tumour development^22,23^. Our research team conducted a comprehensive analysis of tumours from over 3000 patients showing low levels of DARPP-32 expression was associated with poor survival, especially in oestrogen receptor (ER) positive tumours^24–27^. Low *PPP1R1B* mRNA expression was also linked to worse prognosis of patients with ER positive tumours, and *CDC42* and *GRB7*, amongst others, were identified as *PPP1R1B* related genes using Artificial Neural Network (ANN) analysis^24^.

Previous research has shown that transfection of DARPP-32 into the ER positive breast cancer cell line, MCF-7, resulted in reduced cellular migration; however, this effect was not observed in the ER negative breast cancer cell line, MDA-MB-231, that lacked expression of the adhesion receptor DDR1^28^. Co-expression of DARPP-32 and DDR1 in MDA-MD-231 cells resulted in reduced cellular migration, which was dependent on DARPP-32 Thr-34 phosphorylation^28^. Further analysis revealed that cAMP elevation triggered by Wnt-5A at the plasma membrane was responsible for Thr-34 phosphorylation, leading to DARPP-32 dependent inhibition of breast cancer migration^29^.

Emerging evidence indicates that DARPP-32 may play a role in receptor signalling in breast cancer. Specifically, t-DARPP expression in HER2 positive breast cancer cells is linked to trastuzumab resistance, a commonly used HER2 targeted therapy, through increased Bcl-2 expression^30^. This resistance appears to be mediated by t-DARPP phosphorylation at Thr-75, which results in Akt phosphorylation^21^, and subsequent cellular proliferation and growth^23^. Interestingly, co-expression of DARPP-32 can reverse the effect of t-DARPP on trastuzumab resistance and Akt phosphorylation^31^, and the findings have been expanded into oesophageal adenocarcinomas^32^, and with other HER-2 targeted agents, such as lapatinib^33^. In addition, t-DARPP expression can sensitise cells to epidermal growth factor receptor (EGFR) inhibition in the presence of trastuzumab^34^. RNA-sequencing (RNA-Seq) of trastuzumab-resistant breast cancer cells further identified *PPP1R1B* upregulation^35^.

Although research on the link between DARPP-32 and oestrogen is limited, DARPP-32 expression is associated with the survival of ER positive patients^24^, and oestrogen stimulation increases DARPP-32 Thr-34 phosphorylation^36^. In contrast, the role of PKA in oestrogen signalling has been extensively studied. PKA can phosphorylate ERα at Ser-305 to redirect the receptor to new transcriptional start sites, which is associated with tamoxifen resistance in breast cancer; typically, through MYC^37^. Furthermore, indirect links between links between dopamine and oestrogen in the brains of women have been reported (reviewed in^38^), including dopaminergic activation of ER^39^.

The precise mechanism by which DARPP-32 exerts its influence on breast cancer remains poorly understood, nevertheless, current evidence suggests that DARPP-32 may act as a key regulator and/or activator of several critical signalling pathways implicated in cancer development and progression. To shed light on these complex interactions, the current investigation seeks to identify the specific transcriptomic changes that arise following DARPP-32 downregulation in breast cancer.

## Methods

### METABRIC data set

Details of the METABRIC data set (n = 1980) have been published elsewhere^40^. Cohort samples were collected at five centres in the UK and Canada between 1977 and 2005 with appropriate consent from the respective institutional review boards as reported in the original publication. Breast cancer specific survival was calculated as the time interval between primary surgery and death resultant from breast cancer. Almost all ER negative and lymph node positive patients received adjuvant chemotherapy, whereas ER negative and/or lymph node positive patients did not. No patients with HER2 overexpression received trastuzumab. Median follow-up was 141 months determined using the reverse Kaplan–Meier method.

### Cell culture

T47D breast cancer cells were purchased from American Type Culture Collection and were maintained in Dulbecco’s Modified Eagle’s medium with high glucose (Sigma) and supplemented with 10% iron supplemented donor bovine serum (Gibco) and 1% penicillin/streptomycin (Sigma). Cell line identity was verified every 24 months using short tandem repeat (STR) verification and cells were routinely monitored for mycoplasma infection.

### DARPP-32 siRNA knockdown

DARPP-32 expression was knocked down in T47D cells using 8 nM siRNA (OriGene SR313371C) or 8 nM negative control siRNA (OriGene SR30004) and Lipofectamine RNAiMax (Thermo Fisher), in Opti-MEM reduced serum media (Thermo Fisher). For transfection, 3 × 10^6^ cells were allowed to achieve 70–80% confluency over 48 h in T75cm^2^ cell culture flasks. Cell transfection occurred over 24 h, and DARPP-32 knockdown was assessed 24, 48 and 72 h post transfection.

DARPP-32 knockdown was confirmed using Western blotting and PCR. Gel electrophoresis was performed using the Invitrogen Bolt mini system, with 4–12% Bis–Tris plus gels. Lysates were prepared in Bolt LDS sample buffer and Bolt sample reducing buffer and denatured by incubation at 100 °C for five minutes. Transfer to nitrocellulose (Whatman, GE Heathcare) was achieved using Bolt transfer buffer with 10% methanol. Nitrocellulose was blocked in 3% non-fat milk for one hour. Anti-beta-actin (Abcam AB8226, 1:1000) and anti-DARPP-32 (Abcam AB40801, 1:1000) antibodies were incubated on membranes overnight at 4 °C. Secondary antibodies, donkey anti-rabbit immunoglobulin (Li-Cor 926-32213, 1:10000) and donkey anti-mouse immunoglobulin (Li-Cor 926-68072 1:10000) were incubated for one hour prior to visualisation on an Odyssey FC Imager (Li-Cor) using Image Studio software (V4.1). Knockdown of DARPP-32 was also confirmed using PCR, with RNA isolated using RNAprotect Cell Reagent and RNeasy Plus Mini Kit (both Qiagen). Synthesis of cDNA was achieved using RT2 First Strand kit (Qiagen) prior to real-time PCR. Real-time PCR was performed using SYBR Green ROC qPCR Mastermix (Qiagen) and specific primers (DARPP-32 primers (Qiagen 249900), and HPRT primers (forward: 5′-AAATTCTTTGCTGACCTGCTG; reverse: 5′-TCCCCTGTTGACTGGTCATT)) using Viia7TM Real-Time PCR system (Applied Biosystems) operated under Quant StudioTM Real-Time PCR software.

### Cell treatment

Knockdown or control cells were treated with 10 nM 17β-estradiol (E2) (Sigma) dissolved in absolute ethanol for 24 h. E2 treatment was performed in in phenol red free media containing 10% charcoal stripped FBS (Gibco, UK) to remove endogenous serum steroids and eliminate the known weak oestrogen agonistic activity of phenol red. Cells were also treated with 3 µM PKA inhibitor (PKA inhibitor fragment (6–22) amide) (Tocris 1904) dissolved in water, for 24 h. PKA inhibition was performed in Dulbecco’s modified Eagle’s medium with high glucose (Sigma, UK) and 10% iron supplemented Donor Calf Serum (Gibco, UK) without the presence of antibiotics.

The phosphorylation state of DARPP-32, and PKA activity were determined following drug treatment using ELISA and following manufacturer’s instructions (PKA activity ELISA: Thermo Fisher, EIAPKA, DARPP-32 (Thr-34): Assay Genie CBCAB00394, DARPP-32 (Thr-75): Assay Genie CBCAB0010).

### RNA-Seq

RNA extraction, sample quality control, mRNA library preparation and RNA-Seq was performed by Novogene. Briefly, mRNA was purified from total RNA via poly-T oligo attached magnetic beads prior to fragmentation which was followed by first strand cDNA synthesis using random hexamer primers then second strand cDNA synthesis. The library was complete following end-repair, A-tailing, adapter ligation, size selection, amplification and purification. The library was checked with Qubit and real-time PCR for quantification and bioanalyser for size detection before being sequenced on Illumina NovaSeq platforms which utilise a paired-end 150 base pair sequencing strategy. Data output was greater than 20 million read pairs per sample.

### RNA-Seq assessment

Trim Galore version 0.6.7 was used to remove the first 13 base pairs of Illumina standard adaptors (AGATCGGAAGAGC), perform quality trimming (Phred score cutoff 20), and allowed subsequent FastQC analysis. Kallisto was used to quantify the abundance of transcripts from RNA-Seq data using pseudoalignment^41^. The human reference genome was GENCODE GRCh38 version 36 for transcript identification and quantification. The differential expression of transcripts was determined using DESeq2 (1.36.0) in the statistical environment RStudio, relying on input from Kallisto using Tximport. RStudio version 2022.07.1 + 554 running R version 4.2.0, with Bioconductor version 3.15 were used for assessments^42^. The list of transcripts was subject to a multiple test adjustment ranked by a *P* < 0.05 and a greater than two-fold change. Gene enrichment analysis was performed using Qiagen Ingenuity Pathway Analysis (IPA) version 76765844.

### Nottingham patient cohort and immunohistochemistry

Patients with early-stage invasive breast cancer were treated at Nottingham University Hospitals between 1998 and 2006 and underwent wither breast conserving surgery or mastectomy, which was decided by disease characteristics or patient choice, followed by radiotherapy if indicated. Nottingham Prognostic Index (NPI), ER and menopausal status determined if patients received systemic adjuvant treatment. Patients with an NPI score less than 3.4 did not receive adjuvant treatment, and patients with an NPI score of 3.4 and above were candidates for CMF combination chemotherapy (cyclophosphamide, methotrexate and 5-fluorouracil) if they were ER-negative or pre-menopausal; and hormonal therapy if they were ER-positive. No patients received trastuzumab.

Immunohistochemistry was performed on tissue microarrays that were comprised of single 0.6 mm cores taken from representative tumour areas selected by a specialist breast cancer histopathologist from haematoxylin and eosin stained sections. Tissue microarray sections (4 µm) were initially deparaffinised and rehydrated in sequentially in xylene, ethanol and water prior to antigen retrieval in 0.01 mol L^−1^ sodium citrate buffer (pH 6.0), with tissue heated in a microwave for 10 min at 750W, and then 10 min at 450W. Staining was performed using a Novolink Polymer Detection kit (Leica) using the manufacturer’s instructions. Primary antibodies were incubated on tissue for one hour at room temperature (anti-DKK1 1:1000 (Thermo Fisher Scientific, MA5-32229); anti-GRB7 1:500 (Abcam, ab183737); anti-DARPP-32 phosphorylated Thr-34 1:500 (Abcam ab254063)).

Following staining, tissue was dehydrated in ethanol and fixed in xylene prior to mounting using DPX. For each staining run, control breast composite sections comprised of grade 1 and 2 tumours were utilised. Slides were scanned at 20× magnification using a Nanozoomer Digital Pathology Scanner (Hamamatsu Photonics). An immunohistochemical H-score technique was used to assess cytoplasmic staining, whereby the percentage area of tumour staining was classified as 0 to 3, representing, none, weak, intermediate and strong intensity staining. Nuclear staining was scored as the percentage of tumour cells demonstrating any level of staining. Greater than 30% of cores were scored by an independent assessor, with single measure intraclass correlation coefficient values above 0.7 indicating good concordance between scorers.

### Statistics

Statistical analysis was performed using IBM SPSS Statistics (version 28). Cases were stratified based on breast cancer specific survival using X-Tile software and breast cancer specific survival^43^. All differences were deemed statistically significant at the level of *P* ≤ 0.05. The Pearson χ2 test of association was used to determine the relationship between categorised protein expression and clinicopathological variables. Survival curves were plotted according to the Kaplan–Meier method with significance determined using the log-rank test.

### Research involving human participants

Ethical approval for the patient cohort was granted by Nottingham Research Ethics Committee 2, under the title ‘Development of a molecular genetic classification of breast cancer’ (C202313). All procedures performed in studies involving human participants were in accordance with the ethical standards of the institutional and/or national research committee and with the 1964 Helsinki declaration and its later amendments or comparable ethical standards. All samples collected from Nottingham used in this study were pseudo-anonymised; those collected prior to 2006 did not require informed patient consent under the Human Tissue Act, after 2006 informed consent was obtained from all individual participants included in the study.

## Results

### Artificial neural network

ANN analysis was used to identify a stably enriched gene set associated with *PPP1R1B* expression in the METABRIC patient cohort and has been published previously^24^. Three *PPP1R1B* probes were available for assessment and have been described before; briefly, probe 1 and 3 were located in areas found in the sequence for DARPP-32 (NM_032192), and probe 2 and 3 were located in areas found in the sequence for t-DARPP (NM_181505.3)^24^. ANN analysis identified 18 transcripts common to expression of all three available *PPP1R1B* probes within the top 200 transcripts for each probe, including *DKK1* and *GRB7,* which were subject to further investigation.

### DKK1 expression in early-stage breast cancer patients

**Figure 1:**
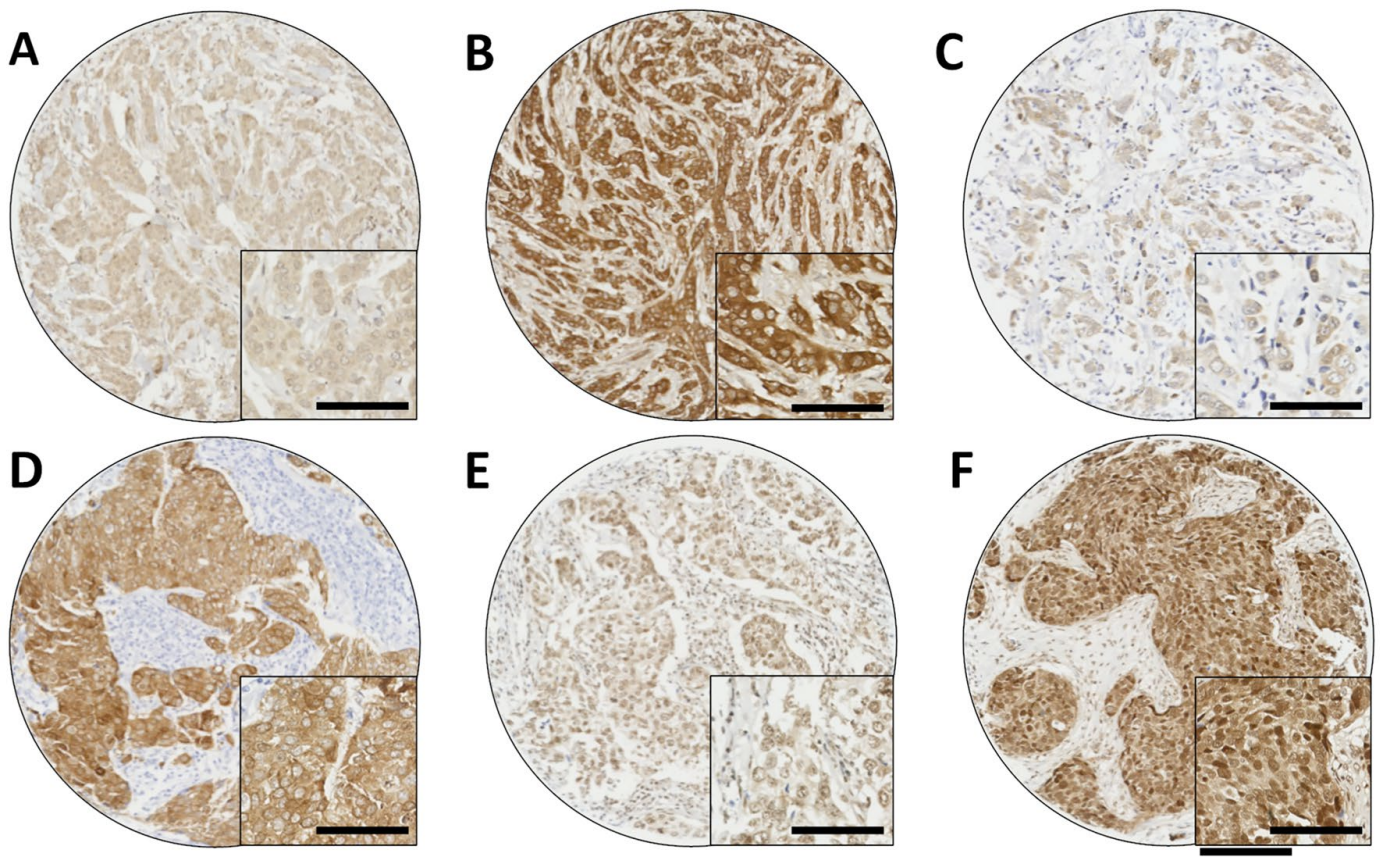
Representative photomicrographs of low DKK1 immunohistochemical staining (**A**), and high staining (**B**); low GRB7 immunohistochemical staining (**C**), and high staining (**D**); low DARPP-32 Thr-34 phosphorylation immunohistochemical staining (**E**), and high staining (**B**). Photomicrographs are shown at 10× magnification with 20× magnification inset box where the scale bar represents 100 µm.

DKK1 protein expression was determined in a cohort of early-stage breast cancer patients. Tissue from 1036 patients were available for assessment, the median H-score for cytoplasmic expression of DKK1 was 110 (ranging between 10 and 260), the median H-score for nuclear DKK1 expression was 25 (ranging between 0 and 100); representative tissue staining is shown in Fig. 1.

**Figure 2:**
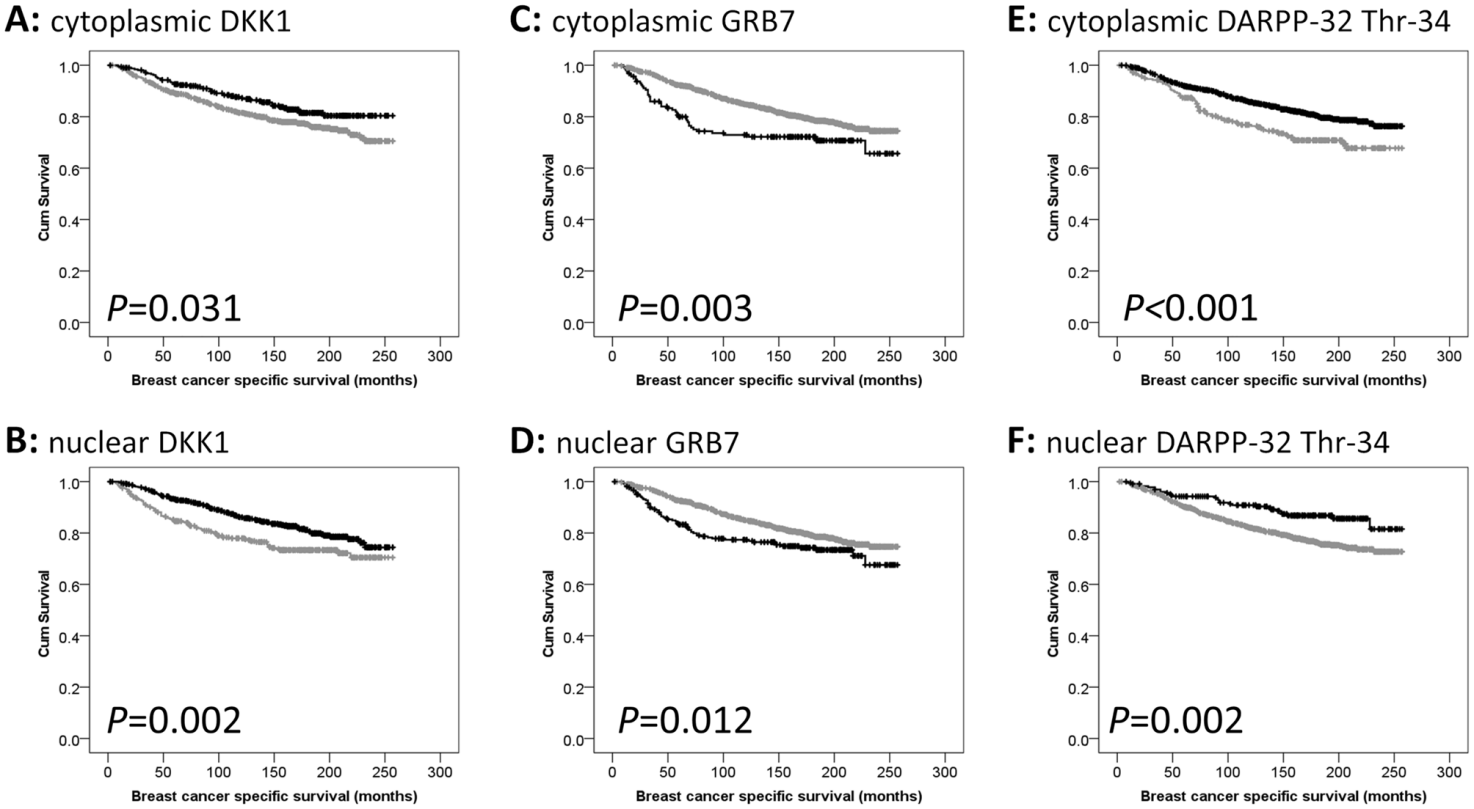
Kaplan–Meier analysis of breast cancer specific survival showing the impact of low (grey line) and high (black line) protein expression: (**A**) DKK1 cytoplasmic expression; (**B**) DKK1 nuclear expression; (**C**) GRB7 cytoplasmic expression; (**D**) GRB7 nuclear expression; (**E**) cytoplasmic DARPP-32 Thr-34 phosphorylation; (**F**) nuclear DARPP-32 Thr-34 phosphorylation.

Low nuclear and cytoplasmic DKK1 expression was significantly associated with adverse breast cancer specific survival (*P* = 0.002, *P* = 0.031 respectively) (Fig. 2). Multivariate analysis was performed using Cox’s proportional hazard method, and included tumour size, tumour grade, tumour stage, NPI, ER status, PgR status, HER2 status and vascular invasion, both nuclear DKK1 and cytoplasmic DKK1 expression was not associated with patient survival in these models (hazard ratio (HR) = 0.834, 95% confidence interval (CI) = 0.625–1.113, *P* = 0.217, and HR = 0.934. 95% CI = 0.667–1.304, *P* = 0.687).

Low levels of DKK1 cytoplasmic expression was significantly associated with larger tumour size (χ^2^ = 5.177, d.f. = 1, *P* = 0.023), higher tumour grade (χ^2^ = 49.148, d.f. = 1, *P* < 0.001), marked nuclear pleomorphism (χ^2^ = 17.603, d.f. = 2, *P* < 0.001), more mitosis (χ^2^ = 49,610, d.f. = 2, *P* < 0.001), intermediate NPI values (χ^2^ = 6.592, d.f. = 2, *P* = 0.037), ER negative tumours (χ^2^ = 66.761, d.f. = 1, *P* = 0.001), negative PgR status (χ^2^ = 45.770, d.f. = 1, *P* < 0.001), HER2 positive tumours (χ^2^ = 6.632, d.f. = 1, *P* = 0.010), triple receptor positive tumours (χ^2^ = 56.570, d.f. = 1, *P* < 0.001), and high Ki67 index (χ^2^ = 21.096, d.f. = 1, *P* < 0.001) at the time of presentation (Table 1). Low nuclear expression of DKK1 was significantly associated with younger patient age (χ^2^ = 7.924, d.f. = 1, *P* = 0.005), larger tumour size (χ^2^ = 6.621, d.f. = 1, *P* = 0.010), high tumour grade (χ^2^ = 14.153, d.f. = 2, *P* < 0.001), marked nuclear pleomorphism (χ^2^ = 27.793, d.f. = 2, *P* < 0.001), mitosis (χ^2^ = 48.622, d.f. = 2, *P* < 0.001), poor NPI values (X^2^ = 41.645, d.f. = 2, *P* < 0.001), ER negative tumours (χ^2^ = 82.423, d.f. = 1, *P* = 0.001), PgR negative tumours (χ^2^ = 33.601, d.f. = 1, *P* < 0.001), triple receptor positive tumours (χ^2^ = 68.76, d.f. = 1, *P* < 0.001), and high Ki67 index (χ^2^ = 16.339, d.f. = 1, *P* < 0.001) at the time of presentation (Table 1).

**Table 1:** Associations between the cytoplasmic and nuclear expression of DKK1 and GRB7 determined using immunohistochemistry with clinicopathological variables.

	Cytoplasmic DKK1 expression			Nuclear DKK1 expression			Cytoplasmic GRB7 expression			Nuclear GRB7 expression		
	Low	High	*P* value	Low	High	*P* value	Low	High	*P* value	Low	High	*P* value
Age												
< 50 years	247 (73.1%)	91 (26.9%)	0.055	137 (40.5%)	201 (59.5%)	**0.005**	371 (84.3%)	69 (15.7%)	** < 0.001**	334 (76.8%)	101 (23.3%)	**0.008**
≥ 50 years	469 (67.2%)	229 (32.8%)		221 (31.7%)	477 (68.3%)		878 (90.7%)	90 (9.3%)		794 (82.8%)	165 (17.2%)	
Tumour Size												
< 2.0 cm	414 (66.5%)	209 (33.5%)	**0.023**	196 (31.5%)	427 (68.5%)	**0.01**	773 (89.4%)	92 (10.6%)	0.326	698 (81.4%)	160 (18.6%)	0.602
≥ 2.0 cm	302 (73.1%)	111 (26.9%)		162 (39.2%)	251 (60.8%)		476 (87.7%)	67 (12.3%)		430 (80.2%)	106 (19.8%)	
Tumour grade												
1	87 (54.4%)	73 (45.6%)	** < 0.001**	34 (21.3%)	126 (78.8%)	** < 0.001**	213 (98.2%)	4 (1.8%)	** < 0.001**	198 (91.7%)	18(8.3%)	** < 0.001**
2	245 (62.3%)	148 (37.7%)		105 (26.7%)	288(73.3%)		537 (95.7%)	24 (4.3%)		495 (89.5%)	58 (10.5%)	
3	384 (79.5%)	99 (25.5%)		219 (45.3%)	264 (54.7%)		499(79.2%)	131 (20.8%)		435 (69.6%)	190 (30.4%)	
Pleomorphism												
1	6 (40.0%)	9 (60.0%)	** < 0.001**	2 (13.3%)	13 (86.7%)	** < 0.001**	22 (100.0%)	0 (0.0%)	** < 0.001**	21 (95.5%)	1 (4.5%)	** < 0.001**
2	175 (61.6%)	109 (38.4%)		68 (23.9%)	216 (76.1%)		395 (97.8%)	9 (2.2%)		364 (91.0%)	36 (9.0%)	
3	535 (72.6%)	202 (27.4%)		288(39.1%)	449 (60.9%)		832 (84.7%)	150 (15.3%)		743 (76.4%)	229 (23.6%)	
Mitosis												
1	280 (58.8%)	196 (41.2%)	** < 0.001**	120 (25.2%)	356 (74.8%)	** < 0.001**	656 (96.9%)	21 (3.1%)	** < 0.001**	605 (90.6%)	63 (9.4%)	** < 0.001**
2	146 (71.6%)	58 (28.4%)		66 (32.4%)	138 (67.6%)		239 (85.7%)	40 (14.3%)		217 (78.3%)	60 (21.7%)	
3	290 (81.5%)	66 (18.5%)		172(48.3%)	184(51.7%)		354 (78.3%)	98 (21.7%)		306 (68.2%)	143(31.8%)	
Vascular Invasion												
Definite	500 (67.6%)	240 (32.4%)	0.089	250 (33.8%)	490 (66.2%)	0.409	915 (90.0%)	102 (10.0%)	**0.016**	832(82.6%)	175 (17.4%)	**0.009**
No/probable	216 (73.0%)	80 (27.0%)		108(36.5%)	188 (63.5%)		334 (85.4%)	57 (14.6%)		296 (76.5%)	91 (23.5%)	
Tumour stage												
1	425 (67.5%)	205 (32.5%)	0.170	207 (32.9%)	423 (67.1%)	0.168	792 (90.4%)	84 (9.6%)	** < 0.001**	724 (83.3%)	145 (16.7%)	** < 0.001**
2	215 (73.4%)	78 (26.6%)		103 (35.2%)	190 (64.8%)		352 (90.0%)	39 (10.0%)		311 (81.0%)	73(19.0%)	
3	75 (67.0%)	37 (33.0%)		47 (42.0%)	65(58.0%)		104 (74.3%)	36 (25.7%)		93 (66.4%)	47 (33.6%)	
NPI												
Good (≤ 3.4)	187 (55.5%)	150 (44.5%)	** < 0.001**	70 (20.8%)	267 (79.2%)	** < 0.001**	471 (97.1%)	14 (2.9%)	** < 0.001**	433 (90.2%)	47 (9.8%)	** < 0.001**
Intermediate (3.41–5.4)	399 (76.1%)	125 (23.9%)		215 (41.0%)	309 (59.0%)		601 (86.2%)	96 (13.8%)		532 (78.2%)	150 (21.8%)	
Poor (> 5.4)	129 (74.1%)	45 (25.9%)		72 (41.4%)	102 (58.6%)		176 (78.2%)	49 (21.8%)		156 (69.6%)	68 (30.4%)	
ER status												
Negative	204 (91.5%)	19 (8.5%)	**0.001**	134 (60.1%)	89 (39.9%)	**0.001**	213 (75.8%)	68 (24.2%)	** < 0.001**	180 (64.7%)	98 (35.3%)	** < 0.001**
Positive	511 (62.9%)	301 (37.1%)		223(27.5%)	589 (72.5%)		1035 (91.9%)	91 (8.1%)		947 (84.9%)	168 (30.2%)	
PgR status												
Negative	346 (80.7%)	83(19.3%)	** < 0.001**	191 (44.5%)	238 (55.5%)	** < 0.001**	462 (80.5%)	112 (19.5%)	** < 0.001**	404 (70.9%)	166 (29.1%)	** < 0.001**
Positive	366 (60.9%)	235 (39.1%)		163 (27.1%)	438 (72.9%)		780 (94.4%)	46 (5.6%)		719 (88.1%)	97 (11.9%)	
HER2 status												
Negative	603 (67.6%)	289 (32.4%)	0.100	299 (32.5%)	593 (66.5%)	0.100	1186(97.1%)	35 (2.9%)	** < 0.001**	1091 (90.3%)	117 (9.7%)	** < 0.001**
Positive	112 (78.3%)	31 (21.7%)		58 (40.6%)	85 (59.4%)		62 (33.3%)	124 (66.7%)		36 (19.5%)	149 (80.5%)	
Triple negative status												
Negative	556 (64.4%)	307 (35.6%)	** < 0.001**	250 (29.0%)	613 (71.0%)	** < 0.001**	1048 (88.1%)	142 (11.9%)	**0.032**	953(80.9%)	225 (19.1%)	0.523
Positive	155 (93.9%)	10 (6.1%)		103 (62.4%)	62 (37.6%)		191 (93.2%)	14 (6.8%)		168 (82.8%)	35 (17.2%)	
Lymph node status												
Negative	425(67.5%)	205 (32.5%)	0.159	207 (32.9%)	423 (67.1%)	0.167	792(90.4%)	84 (9.6%)	**0.009**	724(83.3%)	145 (16.7%)	**0.004**
Positive	290 (71.6%)	115 (28.4%)		150 (37.0%)	225 (63.0%)		456 (85.9%)	75 (14.1%)		404 (77.1%)	120 (22.9%)	
Ki67 index Groups												
Negative	236 (60.7%)	153 (39.3%)	** < 0.001**	100(25.7%)	289 (74.3%)	** < 0.001**	543 (97.8%)	12 (2.2%)	** < 0.001**	498 (91.0%)	49 (9.0%)	** < 0.001**
Positive	292 (76.0%)	92 (24%)		151 (39.9%)	233 (60.7%)		442 (88.9%)	55 (11.1%)		404 (81.9%)	89 (18.1%)	

The *P* values are resultant from Pearson χ^2^ test of association and significant values (*P* < 0.05) are highlighted in bold. ER is oestrogen receptor and PgR is progesterone receptor.

**Figure 3:**
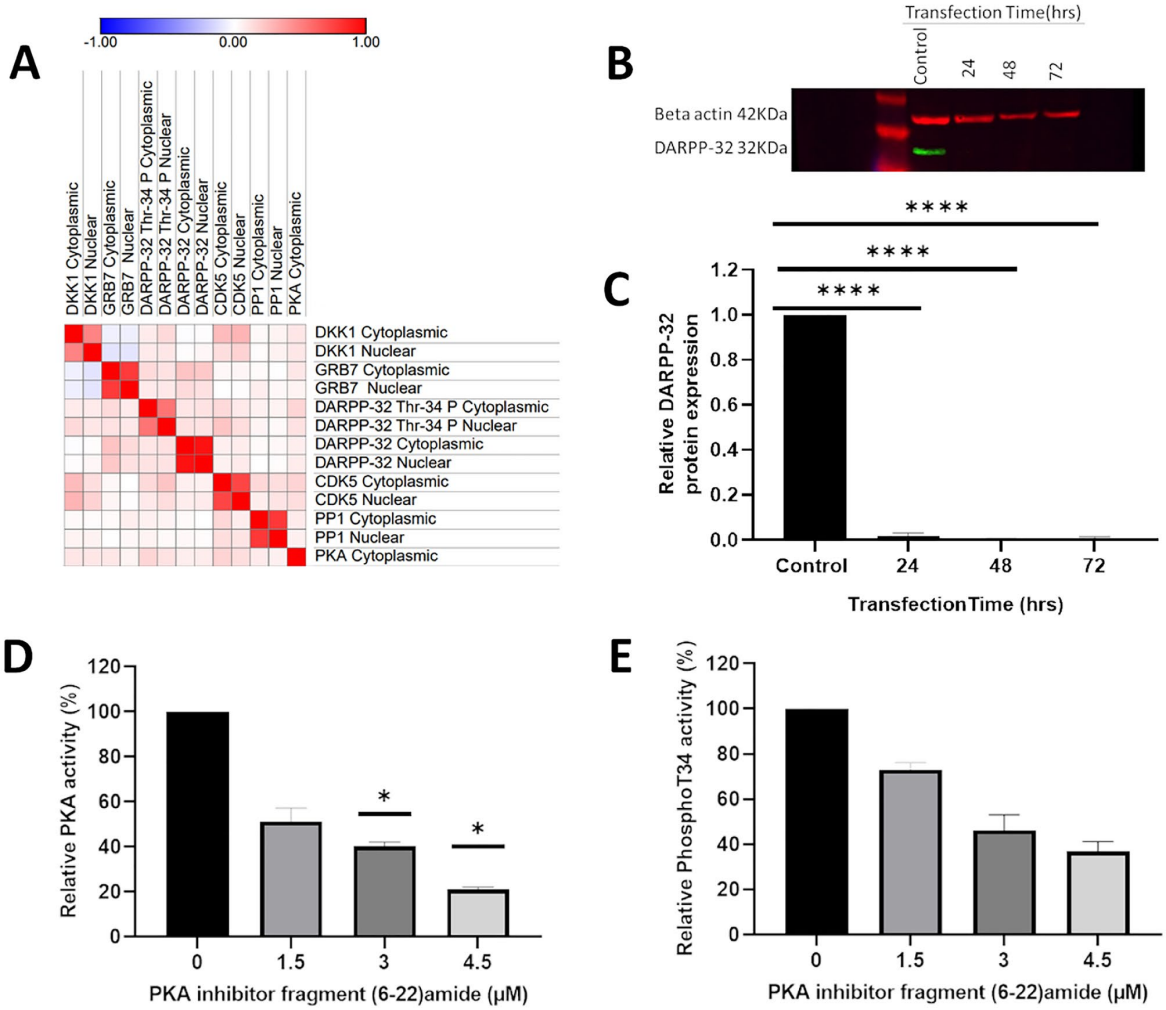
(**A**) correlation matrix demonstrating Spearman Rank Correlations between protein expression of biomarkers determined by immunohistochemistry. (**B**) Western blot assessment of DARPP-32 expression following siRNA DARPP-32 knockdown in T47D breast cancer cells compared against negative control siRNA with uncropped blot available in the Supplementary material. (**C**) real-time PCR of DARPP-32 expression following siRNA DARPP-32 knockdown in T47D breast cancer cells compared against negative control siRNA. (**D**) PKA activity determined by ELISA, and (**E**) DARPP-32 Thr-34 phosphorylation levels determined by ELISA, following inhibition of PKA.

Cytoplasmic DKK1 expression was significantly correlated with DKK1 nuclear expression (R^2^ = 0.483, *P* < 0.001), and DARPP-32 Thr-34 cytoplasmic (R^2^ = 0.079, *P* = 0.015) and nuclear expression (R^2^ = 0.144, *P* < 0.001) phosphorylation. Nuclear DKK1 expression was significantly correlated with GRB7 cytoplasmic (R^2^ = -0.106, *P* < 0.001) and nuclear expression (R^2^ = -0.102, *P* = 0.002), and nuclear DARPP-32 Thr-34 phosphorylation (R^2^ = 0.092, *P* = 0.005). A correlation matrix demonstrating the relationship between DKK1 expression and other variables is shown in Fig. 3A.

### GRB7 expression in early-stage breast cancer patients

GRB7 expression was determined in a cohort of early-stage breast cancer patients. Tissue from 1408 patients were assessed where the median H-score for cytoplasmic expression of GRB7 was 0 (ranging from 0 to 290), the median H-score for nuclear GRB7 expression was 0 (ranging between 0 and 90); representative tissue staining is shown in Fig. 1. A small number of cores did not have a nuclear score assigned due to difficulties in determination. Low nuclear and cytoplasmic expression of GRB7 was significantly associated with good prognosis of breast cancer patients (*P* = 0.012, *P* = 0.003 respectively) (Fig. 2). Multivariate analysis was performed using Cox’s proportional hazard method, and included tumour size, tumour grade, tumour stage, NPI, ER status, PgR status, HER2 status and vascular invasion, both nuclear GRB7 and cytoplasmic GRB7 expression was not associated with patient survival in these models (HR = 0.953, 95% CI = 0.617–1.472, *P* = 0.828, and HR = 1.090. 95% CI = 0.656–1.809, *P* = 0.740).

Low levels of GRB7 cytoplasmic expression was significantly associated with older patient age (χ^2^ = 12.308, d.f. = 1, *P* < 0.001), low tumour grade (χ^2^ = 103.669, d.f. = 2, *P* < 0.001), less nuclear pleomorphism (χ^2^ = 51.487, d.f. = 2, *P* < 0.001), less mitosis (χ^2^ = 96.619, d.f. = 2, *P* < 0.001), absence of vascular invasion (χ^2^ = 5.833, d.f. = 1, *P* = 0.016), low tumour stage (χ^2^ = 32,263, d.f. = 2, *P* =  < 0.001), good NPI value (χ^2^ = 63.142, d.f. = 2, *P* < 0.001), ER positive tumours (χ^2^ = 58.281, d.f. = 1, *P* = 0.001), PgR positive tumours (χ^2^ = 65.761, d.f. = 1, *P* < 0.001), HER2 negative tumours (χ^2^ = 655.473, d.f. = 1, *P* < 0.001), triple receptor positive tumours (χ^2^ = 4.586, d.f. = 1 , *P* = 0.032), negative lymph node status (χ^2^ = 6.784, d.f. = 1, *P* = 0.009) , and low ki67 index (χ^2^ = 34.862, d.f. = 1, *P* < 0.001) at the time of presentation (Table 1). Low nuclear expression of GRB7 was significantly associated with older patient age (χ^2^ = 7.007, d.f. = 1, *P* = 0.008), low tumour grade (χ^2^ = 94.413, d.f. = 2, *P* < 0.001), less nuclear pleomorphism (χ^2^ = 41,964, d.f. = 2, *P* < 0.001), less mitosis (χ^2^ = 88.882, d.f. = 2, *P* < 0.001), absence of vascular invasion (χ^2^ = 6.817, d.f. = 1, *P* = 0.009), low tumour stage (χ^2^ = 22.317, d.f. = 2, *P* < 0.001), good NPI value (χ^2^ = 48.610, d.f. = 1, *P* < 0.001), ER positive tumours (χ^2^ = 58.682, d.f. = 1, *P* < 0.001), PgR positive tumours (χ^2^ = 64.840, d.f. = 1, *P* < 0.001), HER2 negative tumours (χ^2^ = 521,347, d.f. = 1, *P* < 0.001), negative lymph node status (χ^2^ = 6.81, d.f. = 1, *P* = 0.009), and low Ki67 index (χ^2^ = 18.637, d.f. = 1, *P* < 0.001) at the time of presentation (Table 1).

Cytoplasmic GRB7 expression was correlated with nuclear GRB7 expression (R^2^ = 0.768, *P* < 0.001), DKK1 nuclear expression (R^2^ = -0.106, *P* < 0.001) and DARPP-32 Thr-34 cytoplasmic (R^2^ = 0.141, *P* < 0.001) and nuclear (R^2^ = 0.112, *P* < 0.001) phosphorylation. Nuclear GRB7 expression was significantly associated with DKK1 nuclear expression (R^2^ = -0.102, *P* = 0.002) and DARPP-32 Thr-34 cytoplasmic (R^2^ = 0.083, *P* = 0.004) and nuclear (R^2^ = 0.090, *P* = 0.002) phosphorylation. A correlation matrix demonstrating the relationship between GRB7 expression and other variables is shown in Fig. 3A.

### Levels of DARPP-32 Thr-34 phosphorylation in early-stage breast cancer patients

The level of DARPP-32 Thr-34 phosphorylation was determined in a cohort of early-stage breast cancer patients. Tissue from 1274 patients were available for assessment, the median H-score for cytoplasmic expression of DARPP-32 Threonine-34 phosphorylation was 100 (ranging between 0 and 290), the median H-score for nuclear DARPP-32 Threonine-34 phosphorylation expression was 25 (ranging between 0 and 110); representative tissue staining is shown in Fig. 1. Low nuclear and cytoplasmic DARPP-32 Thr-34 phosphorylation was significantly associated with adverse breast cancer specific survival (*P* = 0.002, *P* < 0.001 respectively) (Fig. 2). Multivariate analysis was performed using Cox’s proportional hazard method, and included tumour size, tumour grade, tumour stage, NPI, ER status, PgR status, HER2 status and vascular invasion, both nuclear DARPP-32 Thr-34 phosphorylation remained associated with patient survival in these models (HR = 0.658, 95% CI = 0.443–0.978, *P* = 0.038), but not for cytoplasmic DARPP-32 Thr-34 phosphorylation (HR = 0.763. 95% CI = 0.579–1.008, *P* = 0.057).

Low levels of cytoplasmic DARPP-32 Thr-34 phosphorylation was significantly associated with larger tumour size (χ^2^ = 7.057, d.f. = 1, *P* = 0.008), tumour cell nuclear pleomorphism (χ^2^ = 8.778, d.f. = 2, *P* = 0.012), the presence of vascular invasion (χ^2^ = 5.843, d.f. = 1, *P* = 0.016), higher tumour stage (χ^2^ = 17.966, d.f. = 2, *P* < 0.001), poor NPI values (χ^2^ = 12.216, d.f. = 2, *P* = 0.002), positive lymph node status (χ^2^ = 11.980, d.f. = 1, *P* < 0.001), and low Ki67 index (χ^2^ = 6.671, d.f. = 1, *P* = 0.010) at the time of presentation (Table 2). Low nuclear DARPP-32 Thr-34 phosphorylation was significantly associated with a moderate tumour stage (χ^2^ = 8.644, d.f. = 2, *P* = 0.013), and positive lymph node status (χ^2^ = 8.541, d.f. = 1, *P* = 0.003) at the time of presentation (Table 2).

**Table 2:** Associations between the cytoplasmic and nuclear expression of DARPP-32 Thr-34 phosphorylation determined using immunohistochemistry with clinicopathological variables.

	Cytoplasmic Thr-34 DARPP-32 phosphorylation			Nuclear Thr-34 DARPP-32 phosphorylation		
	Low	High	*P* value	Low	High	*P* value
Age						
< 50 years	87 (20.7%)	334 (79.3%)	0.350	340 (80.8%)	81 (19.2%)	0.477
≥ 50 years	196 (23.0%)	657 (77.0%)		702 (82.4%)	150 (17.6%)	
Tumour size						
< 2.0 cm	152 (19.7%)	619 (80.3%)	**0.008**	625 (81.2%)	145 (18.8%)	0.433
≥ 2.0 cm	131 (26.0%)	372 (74.0%)		417 (82.9%)	86 (17.1%)	
Tumour grade						
1	30 (15.7%)	161(84.3%)	0.064	150 (78.5%)	41 (21.5%)	0.399
2	115 (23.4%)	377 (76.6%)		403 (81.9%)	89 (18.1%)	
3	138 (23.4%)	453 (76.6%)		484 (82.9%)	101 (17.1%)	
Pleomorphism						
1	2 (11.1%)	16 (88.9%)	**0.012**	15 (83.3%)	3 (16.7%)	0.836
2	59 (17.2%)	285 (82.8%)		278 (80.8%)	66(19.2%)	
3	222 (24.3%)	690 (75.5%)		799 (82.2%)	162 (17.8%)	
Mitosis						
1	125(21.3%)	462(78.8%)	0.360	476(81.1%)	111 (18.9%)	0.48
2	67 (25.5%)	196 (74.5%)		222 (84.4%)	41 (15.6%)	
3	91 (21.5%)	333 (78.5%)		344 (81.3%)	79(18.7%)	
Vascular invasion						
Definite	185 (20.4%)	721(79.6%)	**0.016**	731 (80.7%)	175 (19.3%)	0.089
No/probable	98 (26.6%)	270 (73.4%)		311 (84.7%)	56 (15.3%)	
Tumour stage						
1	147 (19.0%)	627 (81.0%)	** < 0.001**	613 (79.3%)	160 (20.7%)	**0.013**
2	90 (24.5%)	277 (75.5%)		316 (86.1%)	51 (13.9%)	
3	46 (34.8%)	86 (65.2%)		112 (84.8%)	20 (15.2%)	
NPI						
Good (≤ 3.4)	77 (18.6%)	337 (81.4%)	**0.002**	325 (78.5%)	89 (21.5%)	0.072
Intermediate (3.41–5.4)	141 (21.8%)	507 (78.2%)		536 (82.8%)	111 (17.2%)	
Poor (> 5.4)	65 (30.8%)	146 (69.2%)		180 (85.3%)	31 (14.7%)	
ER status						
Negative	67 (25.0%)	201 (75.0%)	0.220	221 (82.5%)	47 (17.5%)	0.766
Positive	216 (21.5%)	789 (78.5%)		820 (81.7%)	184 (18.3%)	
PgR status						
Negative	125 (23.9%)	399 (76.1%)	0.276	436 (83.2%)	88 (16.8%)	0.287
Positive	158 (21.3%)	585 (78.7%)		600 (80.9%)	142 (19.1%)	
HER2 status						
Negative	248 (22.5%)	852 (77.5%)	0.496	904 (82.3%)	195 (17.7%)	0.331
Positive	35 (20.2%)	138 (79.8%)		137 (79.2%)	36 (20.8%)	
Triple negative status						
Negative	233 (21.9%)	833 (78.1%)	0.416	868 (81.5%)	197 (18.5%)	0.469
Positive	48 (24.5%)	148 (75.5%)		164 (83.7%)	32 (16.3%)	
Lymph node status						
Negative	147 (19.0%)	627 (81.0%)	** < 0.001**	613 (79.3%)	160 (20.7%)	**0.003**
Positive	136 (27.3%)	363 (72.7%)		428 (85.5%)	71 (14.2%)	
Ki67 index Groups						
Negative	116 (23.3%)	382 (76.7%)	**0.010**	412(82.7%)	86 (17.3%)	0.102
Positive	76 (16.6%)	382 (83.4%)		359 (78.6%)	98 (21.4%)	

The *P* values are resultant from Pearson χ^2^ test of association and significant values (*P* < 0.05) are highlighted in bold. ER is oestrogen receptor and PgR is progesterone receptor.

Correlations between DARPP-32 Thr-34 phosphorylation and DKK1 and GRB7 expression have already been described and shown in Fig. 3A. Cytoplasmic DARPP-32 Thr-34 phosphorylation was significantly correlated with DARPP-32 protein cytoplasmic (R^2^ = 0.098, *P* < 0.001) and nuclear (R^2^ = 0.108, *P* < 0.001) expression. Nuclear DARPP-32 Thr-34 phosphorylation was significantly correlated with DARPP-32 protein cytoplasmic (R^2^ = 0.119, *P* < 0.001) and nuclear (R^2^ = 0.111, *P* < 0.001) expression.

### DARPP-32 knockdown in T47D breast cancer cell line

We sought to identify transcriptomic changes that follow changes in DARPP-32 expression, in the presence and absence of oestrogen and a PKA inhibitor. T47D breast cancer cells were treated with either human DARPP-32 siRNA oligo duplex or negative control siRNA to knockdown DARPP-32 expression. DARPP-32 expression was effectively reduced using siRNA knockdown; protein expression of DARPP-32 was reduced by over 95% determined using Western blotting (Fig. 3B), and over 60% at the mRNA level, using real-time PCR (Fig. 3C); negative control siRNA did not cause a reduction in DARPP-32 expression.

DARPP-32 knockdown cells were also subject to stimulation with E2 or a PKA inhibitor. The PKA inhibitor caused a dose dependent decrease in PKA activity determined by ELISA (Fig. 3D). For RNA-Seq, cells were treated with 3 µM PKA inhibitor for 24 h, which resulted in a 60% reduction in PKA activity, and a 60% reduction in DARPP-32 Thr-34 phosphorylation, which was also determined by ELISA (Fig. 3E).

### RNA-Seq of DARPP-32 knockdown T47D breast cancer cells

When DARPP-32 expression was knocked down in T47D breast cancer cells, 202 differentially expressed transcripts were identified (listed in Supplementary file 1). Following stimulation with E2, 193 differentially expressed transcripts were identified between DARPP-32 knockdown cell treated with E2 versus control cell treated with E2 (listed in Supplementary file 1). When DARPP-32 knockdown T47D cells were treated with a PKA inhibitor and compared with control cells treated with a PKA inhibitor, 181 differentially expressed transcripts were identified (listed in Supplementary file 1). The lists of differentially expressed transcripts were explored for common transcripts between those identified following DARPP-32 knockdown, and then in the presence of E2 or a PKA inhibitor (Table 3A). *PUF60,* and *SART3,* were identified in DARPP-32 knockdown cells and when DARPP-32 knockdown cells were treated with E2. *FIGNL1, TBK1, TSEN34, UBE3A,* and *ZCCHC7* were identified in DARPP-32 knockdown cells, and when DARPP-32 knockdown cells were treated with a PKA inhibitor. *BCLAF1, CAST, CELF1, CTNBB1, KIAA1217 and SMARCE1* were common to DARPP-32 knockdown cells treated with E2 and a PKA inhibitor. *RBM39* and *SLC10A3* were common to all three datasets.

**Table 3:** (A) lists differentially expressed transcripts common to multiple analysis groups, where PKAi is PKA inhibition. (B) lists genes common to both RNA-Seq of DARPP-32 knockdown cells and ANN of *PPP1R1B* probes.

A
Common to DARPP-32 knockdown and DARPP-32 knockdown + E2
* PUF60*
* SART3*
Common to DARPP-32 knockdown and DARPP-32 Knockdown + PKAi
*FIGNL1*
*TBK1*
*TSEN34*
*UBE3A*
*ZCCHC7*
Common to DARPP-32 knockdown treated with E2 or PKAi
*BCLAF1*
*CAST*
*CELF1*
*CTNBB1*
*KIAA1217*
*SMARCE1*
Common to DARPP-32 knockdown, and DARPP-32 knockdown treated with E2 or PKAi
*RBM39*
* SLC10A3*
**B**
Common to RNA-Seq and ANN probe 2
TRAF5
KLK6
GAL3ST4
Common to RNA-Seq and probe 3
LIMCH1
Common to RNA-Seq and ANN probe 2 and 3
PTK7
PPFIBP2
PACSIN2

Qiagen IPA was used to find enriched canonical pathways, 14 were identified when DARPP-32 expression was knocked down. When DARPP-32 knockdown cells were treated with E2, 105 pathways were significantly altered, and 241 when DARPP-32 knockdown cells were treated with a PKA inhibitor (listed in Supplementary file 2). Expectedly dopamine-DARPP-32 feedback in cAMP signaling was one of the 14 pathways identified when DARPP-32 expression was knocked down; the tight junction pathway was common to all three assessments. Qiagen IPA assessment of upstream regulators identified on transcriptional regulator with predicted activation due to changes to target genes in the DARPP-32 knockdown dataset. Changes to *CBFA2T3*, *CEMIP2*, *KDM3A*, *MTF2* and *NOTCH4* expression indicated activation of the upstream regulator *SOX2*.

### RNA-Seq assessment and commonalities with Artificial Neural Network analysis of METABRIC cohort

The significant differentially expressed transcripts identified through RNA-Seq of DARPP-32 knockdown T47D breast cancer cells were compared with those identified as associated with PPP1R1B expression in the METABRIC patient cohort using ANN analysis to find common genes. Of the 202 transcripts identified using RNA-Seq, seven of those were common to the top 300 genes identified using ANN analyses (Table 3B). All seven of the common genes were identified in gene lists from the ANN of *PPP1R1B* probes 2 and 3. *PTK7*, *PPFIBP2* and *PACSIN2* were identified in the ANN of *PPP1R1B* probes 2 and 3, whilst *TRAF5*, *KLK6*, *GAL3ST4* were identified within the *PPP1R1B* probe 2 analysis, and *LIMCH1* was identified within the *PPP1R1B* probe 3 analysis.

Common genes were also identified between the expression of *PPP1R1B* probes and DARPP-32 knockdown cells that were treated with E2 and that were treated with a PKA inhibitor. *EHMT2, KCNIP2, GOLGA2,* and *ADCY1,* were identified in the ANN of *PPP1R1B* probe 1 and when DARPP-32 knockdown cells were treated with E2. *GSTCD* was identified in the ANN of *PPP1R1B* probe 2 and when DARPP-32 knockdown cells were treated with E2*. EPHB6,* and *VASN* were identified in the ANN of *PPP1R1B* probe 3 and when DARPP-32 knockdown cells were treated with E2.

No common genes were identified in the ANN of *PPP1R1B* probe 1 when DARPP-32 knockdown cells were treated with a PKA inhibitor. *BMPR1B,* and *MTA1* were identified in the ANN of *PPP1R1B* probe 2 when DARPP-32 knockdown cells were treated with a PKA inhibitor and *ARL6IP4* was identified in the ANN of probe 3.

*PAQR6* was identified in the ANN of *PPP1R1B* probe 1 and probe 2 and when DARPP-32 knockdown cells were treated with E2. No genes were identified that were common to all three *PPP1R1B* probes between the three datasets.

## Conclusion

In a previous study, we established an association between low DARPP-32 expression and poor prognosis of breast cancer patients, particularly those with ER positive tumours^24^. However, the underlying mechanism by which DARPP-32 impacts breast cancer cell behaviour remains unknown. Using an ANN analysis we identified 18 transcripts common to expression of all three available *PPP1R1B* probes within the top 200 transcripts for each probe, including *DKK1* and *GRB7*. To further investigate this finding, we conducted a large-scale cohort study to determine GRB7 and DKK1 protein expression levels in combination with determining levels of DARPP-32 Thr-34 phosphorylation in the same patient specimens. In this study we utilised the level of DARPP-32 Thr-34 phosphorylation as a proxy for full-length DARPP-32 expression. DARPP-32 Thr-34 can act as a surrogate of full length DARPP-32 phosphorylation as t-DARPP is lacking this residue; unfortunately, DARPP-32 Thr-75 phosphorylation could not be determined with sufficient accuracy to warrant further study. Our results show that low levels of DARPP-32 Thr-34 phosphorylation was significantly associated with poor patient prognosis, which was in direct alignment with our previous findings^24^. Notably, this association remains significant for nuclear DARPP-32 Thr-34 phosphorylation in multivariate survival analysis. Additionally, DARPP-32 Thr-34 phosphorylation was significantly associated with advanced tumour stage and lymph node involvement. This study provides further validation that DARPP-32 may be a clinically relevant biomarker in breast cancer.

DKK1 is a secretory antagonist of the classical Wnt signalling pathway; studies have indicated that dysregulation of the Wnt signalling pathway, induced via the activity of DKK1, is important to cancer cell migration and bone metastasis in lung, breast and prostate cancer^44–46^. Contrasting results demonstrate that whilst overexpression of DKK1 is linked with migration and invasion of hepatocellular carcinoma, DKK1 inhibits migration and invasion of colon and breast cancer^47–49^. In this study, low expression of DKK1 was significantly associated with adverse breast cancer specific survival. In addition, low levels of DKK1 expression associated with larger tumours, higher tumour grade, and ER and PgR negative tumours. As expected, we observed a weak but statistically significant correlation between DKK1 expression and DARPP-32 Thr-34 phosphorylation.

GRB7 is a 532 amino acid adaptor molecule with a crucial role in the activation of multiple intracellular pathways through transmission of signals from cell membrane receptors. In this study low nuclear and cytoplasmic expression of GRB7 was significantly associated with good prognosis of breast cancer patients. In addition, low levels of GRB7 expression were associated with lower tumour grade, tumour stage, ER and PgR positive tumours and HER2 negative tumours. This is aligned with previous studies that have demonstrated that low GRB7 expression is associated with improved survival of breast cancer patients (n = 638)^50^ and GRB7 is included in the 21 gene set of Oncotype DX. As expected, we observed a weak but statistically significant correlation between GRB7 expression and DARPP-32 Thr-34 phosphorylation. An association between *PPP1R1B* and *GRB7* has been previously demonstrated in upper gastrointestinal adenocarcinomas^51^.

Using the ER positive breast cancer cell line, T47D, we sought to identify transcriptomic changes that follow changes in DARPP-32 expression, in the presence and absence of oestrogen and a PKA inhibitor. The actions of E2, or 17β-estradiol, are mediated by ER, and treatment of T47D cells with 10 nM E2, has been shown to increase cellular proliferation with significant changes in the transcriptome, including the expression of important cancer associated genes, such as *MYC*^52–55^. PKA inhibition with PKA inhibitor fragment (6–22) amide was used determine transcriptomic changes that occur following reduction of PKA mediated DARPP-32 Thr-34 phosphorylation; however, PKA inhibition would also be expected to alter a wide range of cell signalling effects directly through PKA. PKA inhibitor fragment (6–22) amide is a potent inhibitor of PKA that is derived from the active portion of the heat stable PKA inhibitor protein, PKI. In T47D cells, 24-h 3 µM PKA inhibitor treatment resulted in a 60% inhibition of PKA, and a 60% decrease in DARPP-32 Thr-34 phosphorylation. 202 differentially expressed transcripts were identified following knockdown of DARPP-32. Following stimulation with E2, 193 differentially expressed transcripts were identified and 181 following treatment with a PKA inhibitor. Transcripts common to multiple assessments were identified, with *PUF60*, and *SART3* common to DARPP-32 knockdown cells and DARPP-32 knockdown cells treated with E2. *FIGNL1*, *TBK1*, *TSEN34*, *UBE3A*, and *ZCCHC7* were common to DARPP-32 knockdown cells and DARPP-32 knockdown cells treated with a PKA inhibitor. *RBM39* and *SLC10A3* were common to all three DARPP-32 knockdown RNA-Seq data sets; *RBM39* encodes RNA binding motif protein 39 (RBM39), and *SLC10A3* encodes solute carrier family 10 member 3.

Transcripts common to multiple assessments included a number of genes linked to breast cancer. Expression of *PUF60*, a spliceosome component, is associated with overall survival in breast cancer^56^, and *SART3* has previously been identified as expressed in a large proportion of breast cancer cell tissue^57^. Serum levels of TBK1, are associated with the clinical outcome of breast cancer patients^58^ and ubiquitin protein ligase E3A (*UBE3A*) has been shown to be overexpressed in breast cancer^59^. *RBM39* and *SLC10A3* were common to all datasets and have been linked with tumour cell behaviour^60^ and high expression associated with adverse survival of hepatocellular cancer patients^61^, respectively.

The transcripts identified through RNA-Seq of DARPP-32 knockdown T47D breast cancer cells were compared with those identified as associated with *PPP1R1B* expression in the METABRIC patient cohort. Seven genes were identified that were common between lists, with all identified in ANN gene lists from *PPP1R1B* probe 2 and 3. *PTK7*, *PPFIBP2* and *PACSIN2* were identified in the ANN of *PPP1R1B* probes 2 and 3, *TRAF5*, *KLK6*, *GAL3ST4* were identified within the *PPP1R1B* probe 2 analysis, and *LIMCH1* was identified within the *PPP1R1B* probe 3 analysis. Common genes were also identified between the METABRIC ANN and in DARPP-32 knockdown cells treated with E2 and the PKA inhibitor. Of these genes, *PTK7*, *KLK6* and *LIMCH1* have interesting links with breast cancer; *PTK7* is a catalytically inactive receptor tyrosine kinase in the Wnt signalling pathway, and a PTK7 targeted antibody–drug conjugate has been shown to reduce tumour-initiating cells^62^. Both *KLK6* and *LIMCH1* expression has been linked to clinical outcome of breast cancer patients^63,64^.

The significant associations observed between DARPP-32 and its downstream targets DKK1 and GRB7 and patient survival, underscore their importance in breast cancer. By using an approach that combines patient and cell line transcriptomics we have identified a number of common genes that provides an insight into the molecular mechanisms underlying differential breast cancer prognosis and highlights potential targets for therapeutic intervention.

### Supplementary Information

Supplementary Information 1.

Supplementary Information 2.

Supplementary Figures.

## Data Availability

The data have been deposited in NCBI’s Gene Expression Omnibus (GEO) and are accessible through GEO Series accession number GSE204836 (https://www.ncbi.nlm.nih.gov/geo/query/acc.cgi?acc=GSE204836). The METABRIC data is publicly available https://www.ebi.ac.uk/ega/studies/EGAS00000000098. Code related to the analysis can be found on github at https://github.com/sarahstorr.

## References

[1] Hemmings HC, Nairn AC, Aswad DW, Greengard P (1984). DARPP-32, a dopamine- and adenosine 3′:5′-monophosphate-regulated phosphoprotein enriched in dopamine-innervated brain regions. II. Purification and characterization of the phosphoprotein from bovine caudate nucleus. J. Neurosci..

[2] Ouimet CC, Miller PE, Hemmings HC, Walaas SI, Greengard P (1984). DARPP-32, a dopamine- and adenosine 3':5'-monophosphate-regulated phosphoprotein enriched in dopamine-innervated brain regions. III. Immunocytochemical localization. J. Neurosci..

[3] Walaas SI, Greengard P (1984). DARPP-32, a dopamine- and adenosine 3′:5′-monophosphate-regulated phosphoprotein enriched in dopamine-innervated brain regions. I. Regional and cellular distribution in the rat brain. J. Neurosci..

[4] Hemmings HC, Greengard P, Tung HY, Cohen P (1984). DARPP-32, a dopamine-regulated neuronal phosphoprotein, is a potent inhibitor of protein phosphatase-1. Nature.

[5] Hemmings HC, Nairn AC, Greengard P (1984). DARPP-32, a dopamine- and adenosine 3':5'-monophosphate-regulated neuronal phosphoprotein. II. Comparison of the kinetics of phosphorylation of DARPP-32 and phosphatase inhibitor 1. J. Biol. Chem..

[6] Fienberg AA, Greengard P (2000). The DARPP-32 knockout mouse. Brain Res. Brain Res. Rev..

[7] Greener MR, Storr SJ (2022). Exploring the role of DARPP-32 in addiction: A review of the current limitations of addiction treatment pathways and the role of DARPP-32 to improve them. NeuroSci.

[8] Bibb JA (1999). Phosphorylation of DARPP-32 by Cdk5 modulates dopamine signalling in neurons. Nature.

[9] El-Rifai W (2002). Gastric cancers overexpress DARPP-32 and a novel isoform, t-DARPP. Cancer Res..

[10] Beckler A (2003). Overexpression of the 32-kilodalton dopamine and cyclic adenosine 3',5'-monophosphate-regulated phosphoprotein in common adenocarcinomas. Cancer.

[11] Wang MS (2005). Overexpression of DARPP-32 in colorectal adenocarcinoma. Int. J. Clin. Pract..

[12] Ebihara Y (2004). DARPP-32 expression arises after a phase of dysplasia in oesophageal squamous cell carcinoma. Br. J. Cancer.

[13] Alam SK (2020). ASCL1-regulated DARPP-32 and t-DARPP stimulate small cell lung cancer growth and neuroendocrine tumour cell proliferation. Br. J. Cancer.

[14] Alam SK (2022). DARPP-32 promotes ERBB3-mediated resistance to molecular targeted therapy in EGFR-mutated lung adenocarcinoma. Oncogene.

[15] Alam SK, Wang L, Zhu Z, Hoeppner LH (2023). IKKalpha promotes lung adenocarcinoma growth through ERK signaling activation via DARPP-32-mediated inhibition of PP1 activity. NPJ. Precis. Oncol..

[16] Alam SK (2018). DARPP-32 and t-DARPP promote non-small cell lung cancer growth through regulation of IKKalpha-dependent cell migration. Commun. Biol..

[17] Hong L (2007). DARPP-32 mediates multidrug resistance of gastric cancer through regulation of P-gp and ZNRD1. Cancer Invest..

[18] Belkhiri A, Dar AA, Zaika A, Kelley M, El-Rifai W (2008). t-Darpp promotes cancer cell survival by up-regulation of Bcl2 through Akt-dependent mechanism. Cancer Res..

[19] Belkhiri A, Zhu S, Chen Z, Soutto M, El-Rifai W (2012). Resistance to TRAIL is mediated by DARPP-32 in gastric cancer. Clin. Cancer Res..

[20] Zhu S (2013). Regulation of CXCR4-mediated invasion by DARPP-32 in gastric cancer cells. Mol. Cancer Res..

[21] Hamel S (2010). Both t-Darpp and DARPP-32 can cause resistance to trastuzumab in breast cancer cells and are frequently expressed in primary breast cancers. Breast Cancer Res. Treat..

[22] Christenson JL, Kane SE (2014). Darpp-32 and t-Darpp are differentially expressed in normal and malignant mouse mammary tissue. Mol. Cancer.

[23] Vangamudi B (2010). t-DARPP regulates phosphatidylinositol-3-kinase-dependent cell growth in breast cancer. Mol. Cancer.

[24] Kotecha S (2019). Dopamine and cAMP-regulated phosphoprotein 32 kDa (DARPP-32) and survival in breast cancer: a retrospective analysis of protein and mRNA expression. Sci. Rep..

[25] Martin SG (2020). Dopamine and cAMP-regulated phosphoprotein 32kDa (DARPP-32), protein phosphatase-1 and cyclin-dependent kinase 5 expression in ovarian cancer. J. Cell Mol. Med..

[26] Saidy B (2021). PP1, PKA and DARPP-32 in breast cancer: A retrospective assessment of protein and mRNA expression. J. Cell Mol. Med..

[27] Saidy B (2020). Retrospective assessment of cyclin-dependent kinase 5 mRNA and protein expression and its association with patient survival in breast cancer. J. Cell Mol. Med..

[28] Hansen C, Greengard P, Nairn AC, Andersson T, Vogel WF (2006). Phosphorylation of DARPP-32 regulates breast cancer cell migration downstream of the receptor tyrosine kinase DDR1. Exp. Cell Res..

[29] Hansen C (2009). Wnt-5a-induced phosphorylation of DARPP-32 inhibits breast cancer cell migration in a CREB-dependent manner. J. Biol. Chem..

[30] Belkhiri A (2008). Expression of t-DARPP mediates trastuzumab resistance in breast cancer cells. Clin. Cancer Res..

[31] Gu L, Waliany S, Kane SE (2009). Darpp-32 and its truncated variant t-Darpp have antagonistic effects on breast cancer cell growth and herceptin resistance. PLoS One.

[32] Hong J (2012). Regulation of ERBB2 receptor by t-DARPP mediates trastuzumab resistance in human esophageal adenocarcinoma. Cancer Res..

[33] Christenson JL, Denny EC, Kane SE (2015). t-Darpp overexpression in HER2-positive breast cancer confers a survival advantage in lapatinib. Oncotarget.

[34] Denny EC, Kane SE (2015). t-Darpp promotes enhanced EGFR activation and new drug synergies in Her2-positive breast cancer cells. PLoS One.

[35] Murad R (2021). Transcriptome and chromatin landscape changes associated with trastuzumab resistance in HER2+ breast cancer cells. Gene.

[36] Auger AP, Meredith JM, Snyder GL, Blaustein JD (2001). Oestradiol increases phosphorylation of a dopamine- and cyclic AMP-regulated phosphoprotein (DARPP-32) in female rat brain. J. Neuroendocrinol..

[37] de Leeuw R (2013). PKA phosphorylation redirects ERalpha to promoters of a unique gene set to induce tamoxifen resistance. Oncogene.

[38] Del Rio JP (2018). Steroid hormones and their action in women's brains: The importance of hormonal balance. Front. Public Health.

[39] Olesen KM, Auger AP (2008). Dopaminergic activation of estrogen receptors induces fos expression within restricted regions of the neonatal female rat brain. PLoS One.

[40] Curtis C (2012). The genomic and transcriptomic architecture of 2000 breast tumours reveals novel subgroups. Nature.

[41] Bray NL, Pimentel H, Melsted P, Pachter L (2016). Near-optimal probabilistic RNA-seq quantification. Nat. Biotechnol..

[42] Anders S, Huber W (2010). Differential expression analysis for sequence count data. Genome Biol..

[43] Camp RL, Dolled-Filhart M, Rimm DL (2004). X-tile: A new bio-informatics tool for biomarker assessment and outcome-based cut-point optimization. Clin. Cancer Res..

[44] Pang H (2018). Effects of DKK1 overexpression on bone metastasis of SBC-3 cells. Oncol. Lett..

[45] Thudi NK (2011). Dickkopf-1 (DKK-1) stimulated prostate cancer growth and metastasis and inhibited bone formation in osteoblastic bone metastases. Prostate.

[46] Bu G (2008). Breast cancer-derived Dickkopf1 inhibits osteoblast differentiation and osteoprotegerin expression: Implication for breast cancer osteolytic bone metastases. Int. J. Cancer.

[47] Chen L, Li M, Li Q, Wang CJ, Xie SQ (2013). DKK1 promotes hepatocellular carcinoma cell migration and invasion through beta-catenin/MMP7 signaling pathway. Mol. Cancer.

[48] Gonzalez-Sancho JM (2005). The Wnt antagonist DICKKOPF-1 gene is a downstream target of beta-catenin/TCF and is downregulated in human colon cancer. Oncogene.

[49] Niu J (2019). DKK1 inhibits breast cancer cell migration and invasion through suppression of beta-catenin/MMP7 signaling pathway. Cancer Cell Int..

[50] Nadler Y (2010). Growth factor receptor-bound protein-7 (Grb7) as a prognostic marker and therapeutic target in breast cancer. Ann. Oncol..

[51] Maqani N (2006). Molecular dissection of 17q12 amplicon in upper gastrointestinal adenocarcinomas. Mol. Cancer Res..

[52] Dubik D, Dembinski TC, Shiu RP (1987). Stimulation of c-myc oncogene expression associated with estrogen-induced proliferation of human breast cancer cells. Cancer Res..

[53] Katchy A, Edvardsson K, Aydogdu E, Williams C (2012). Estradiol-activated estrogen receptor alpha does not regulate mature microRNAs in T47D breast cancer cells. J. Steroid Biochem. Mol. Biol..

[54] Strom A (2004). Estrogen receptor beta inhibits 17beta-estradiol-stimulated proliferation of the breast cancer cell line T47D. Proc. Natl. Acad. Sci. U S A.

[55] Yu S, Kim T, Yoo KH, Kang K (2017). The T47D cell line is an ideal experimental model to elucidate the progesterone-specific effects of a luminal A subtype of breast cancer. Biochem. Biophys. Res. Commun..

[56] An J (2021). Identification of spliceosome components pivotal to breast cancer survival. RNA Biol..

[57] Suefuji Y (2001). Expression of SART3 antigen and induction of CTLs by SART3-derived peptides in breast cancer patients. Br. J. Cancer.

[58] Liu H, Li H, Zhang J, Meng Q, Ma L (2022). Correlation of TBK1, AR, and other serum cancer-related biomarkers in breast cancer patients: An observational study. Medicine (Baltimore).

[59] Deng S (2007). Over-expression of genes and proteins of ubiquitin specific peptidases (USPs) and proteasome subunits (PSs) in breast cancer tissue observed by the methods of RFDD-PCR and proteomics. Breast Cancer Res. Treat..

[60] Puvvula PK (2021). Inhibiting an RBM39/MLL1 epigenomic regulatory complex with dominant-negative peptides disrupts cancer cell transcription and proliferation. Cell Rep..

[61] Tian S, Li J, Xiang J, Peng P (2022). The clinical relevance and immune correlation of SLC10 family genes in liver cancer. J. Hepatocell. Carcinoma.

[62] Damelin M (2017). A PTK7-targeted antibody-drug conjugate reduces tumor-initiating cells and induces sustained tumor regressions. Sci. Transl. Med..

[63] Alifanov VV, Tashireva LA, Zavyalova MV, Perelmuter VM (2022). LIMCH1 as a new potential metastasis predictor in breast cancer. Asian Pac. J. Cancer Prev..

[64] Haritos C (2018). Kallikrein-related peptidase 6 (KLK6) expression differentiates tumor subtypes and predicts clinical outcome in breast cancer patients. Clin. Exp. Med..

