# Supplementary figures and images for: Unravelling transcriptomic complexity in breast cancer through modulation of DARPP-32 expression and signalling pathways
Source: Sci Rep. 2023 Nov 30;13:21163. doi: 10.1038/s41598-023-48198-y (PMC10689788; doi:10.1038/s41598-023-48198-y)

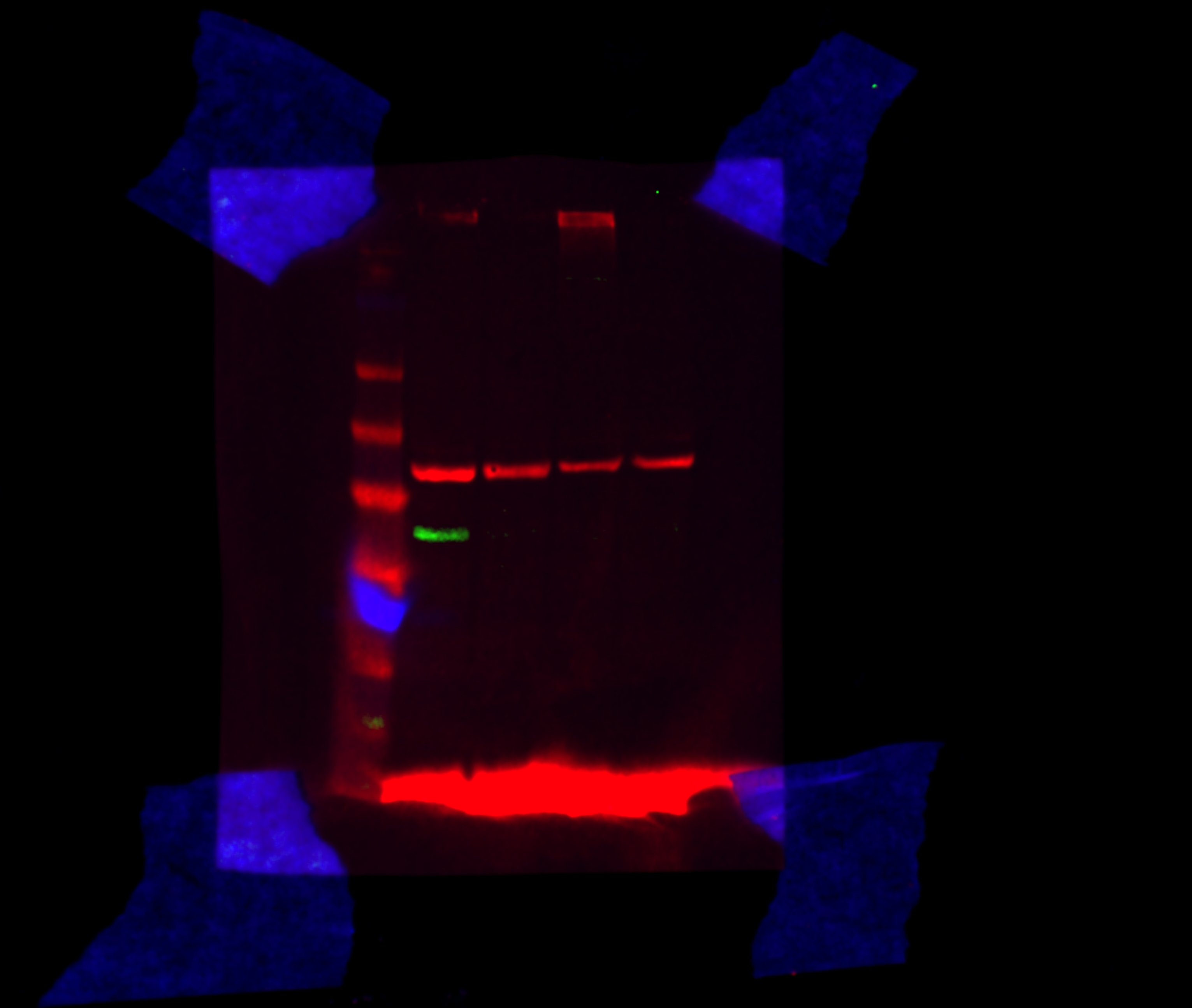

Supplement: Supplementary file 3 — Supplementary Figures. [file 41598_2023_48198_MOESM3_ESM.jpg]
